# Increased levels of NT-proBNP and troponin T 2 years after coronary artery bypass grafting complicated by mediastinitis

**DOI:** 10.3389/fcvm.2023.1008825

**Published:** 2023-02-07

**Authors:** Ivar Risnes, Pål Aukrust, Runar Lundblad, Olaf Rødevand, Thor Ueland, Stein Erik Rynning, Sahrai Saeed

**Affiliations:** ^1^Department of Cardiac Surgery, LHL Heart Clinic, Gardermoen, Norway; ^2^Department of Thoracic and Cardiovascular Surgery, Oslo University Hospital, Rikshospitalet, Oslo, Norway; ^3^Department of Heart Disease, Haukeland University Hospital, Bergen, Norway; ^4^Research Institute of Internal Medicine, Oslo University Hospital, Rikshospitalet, Oslo, Norway; ^5^Institute of Clinical Medicine, Faculty of Medicine, University of Oslo, Oslo, Norway; ^6^Section of Clinical Immunology and Infectious Diseases, Oslo University Hospital, Rikshospitalet, Oslo, Norway

**Keywords:** cardiac biomarkers in mediastinitis following coronary artery bypass grafting, coronary artery bypass grafting, C-reactive protein, mediastinitis, N-terminal probrain natriuretic peptide, troponin T

## Abstract

**Background:**

Mediastinitis after coronary bypass grafting (CABG) increases the risk of the internal mammary artery (IMA) graft obstruction, and has a detrimental effect on long-term survival. The pathogenesis for this increased mortality is poorly understood. In the present study, we aimed to investigate the relationship between mediastinitis and persistently elevated cardiac-specific biomarkers [troponin T (TnT) and N-terminal pro-brain natriuretic peptide (NT-proBNP)] and C-reactive protein (CRP) at mid-term follow-up following CABG.

**Material and methods:**

The epidemiologic design was of an exposed (mediastinitis, *n* = 41) vs. randomly selected non-exposed (non-mediastinitis) controls (*n* = 41) cohort. Serum samples for measurements of NT-proBNP, TnT, and CRP were obtained at a median follow up time of 2.7 (range 0.5–5.2) years after CABG surgery.

**Results:**

NT-proBNP (mean 65.0 pg/ml vs. 34.8 pg/ml, *p* = 0.007) and TnT levels (mean 14.7 ng/L vs. 11.2 ng/L, *p* = 0.004) were significantly higher in the mediastinitis group than in the control group. Patients with mediastinitis had also higher body mass index (BMI) and were more likely to have diabetes and previous myocardial infarction. There was no difference in serum CRP level between the groups. After controlling for potential confounders (previous myocardial infarction, age, and BMI), the presence of mediastinitis was associated with higher levels of log NT-proBNP (*p* = 0.02) and log TnT (*p* = 0.04).

**Conclusion:**

Mediastinitis increases the concentrations of cardiac-specific biomarkers NT-proBNP and TnT at mid-term follow-up, representing persistent myocardial injury and impaired cardiac function.

## Introduction

The incidence of mediastinitis after coronary artery bypass grafting (CABG) is low; around 1%. However, this feared complication is associated with an increased risk of morbidity and decreased long-term survival (1–4). In addition, the risk of cardiac death is significantly higher in patients with mediastinitis compared to patients without mediastinitis (1). The mechanism for development of mediastinitis and its complications is multifactorial, and not fully understood (1–5). Mediastinitis increases the risk of early internal mammary artery (IMA) graft obstruction (6), which may be due to inflammation or mechanical damage to the IMA. IMA graft failure is probably one of several factors that explains cardiac death after mediastinitis. N-terminal pro-brain natriuretic peptide (NT-proBNP) is synthesized and released from the myocardium in response to increased wall stress and injury (7, 8). The serum levels of NT-proBNP are increased in patients with heart failure (HF), and have been shown to provide important prognostic information in these patients (9, 10). Similarly, higher concentrations of troponin T (TnT), a specific marker of cardiomyocyte damage, have been shown to be prognostically important, not only in patients with myocardial infarction (MI) and unstable coronary artery disease (CAD), but also in patients with stable CAD and in patients with HF (11, 12).

C-reactive protein (CRP) is a stable and reliable marker of inflammation, and has been shown to give prognostic information in a wide number of atherosclerotic disorders, as well as HF, reflecting the involvement of inflammatory pathways in these disorders (13, 14). Numerous studies have shown that NT-proBNP, TnT and CRP provide important prognostic information in various cardiovascular disorders. However, whether these markers are regulated by mediastinitis following CABG, is not fully elucidated. Therefore, in the present study, we examined the levels of NT-proBNP, TnT and CRP following CABG in patients with and without complicating mediastinitis.

## Materials and methods

### Study population

Between September 2005 and April 2010, a total of 6,620 patients >18 years undergoing CABG surgery were considered as the source population for the present study. In this period, patients suffering from deep sternal wound infection were treated with vacuum-assisted closure (VAC). The diagnosis was based upon the criteria established by the Centres for Disease Control and Prevention (www.cdc.gov) (15, 16).

### Epidemiological design

**Figure 1:**
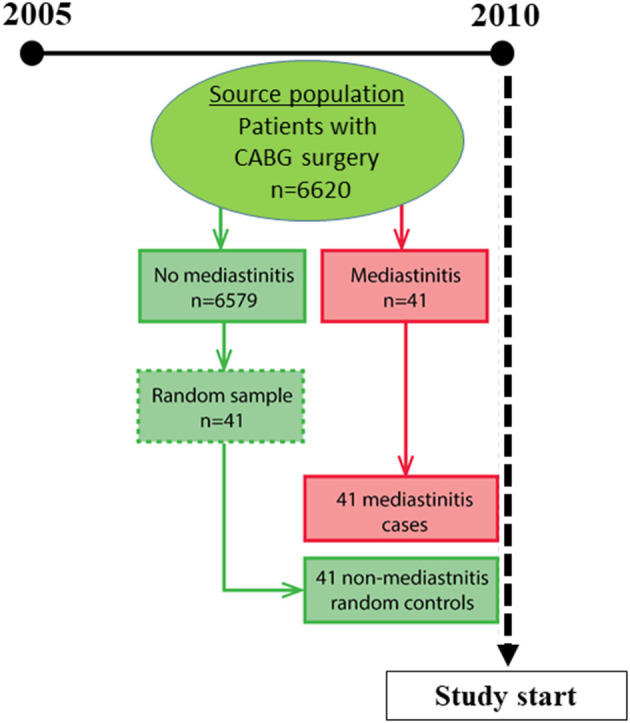
Study design.

This is a cohort study of 82 patients undergoing CABG surgery. By epidemiological design, the study included 41 exposed (mediastinitis) and 41 non-exposed (non-mediastinitis), controls. Blood samples were collected and analyzed for; (1) NT-proBNP, (2) TnT and (3) CRP. Blood samples were obtained at routine examination at a median follow up time of 2.7 (range, 0.5–5.2) years after primary CABG surgery. All CABG patients who had developed post-operative mediastinitis during 4.7 years follow-up, from September 2005 to April 2010, were considered as exposed patients. The non-exposed cohort was a random control sample of 41 patients without mediastinitis (Figure 1), collected from the same source population and the same period of time (17). Evaluation of the laboratory markers was performed during a period of nine months, from April to December 2010. Median observation time from primary CABG to laboratory control and coronary CT angiography was 2.6 (range 0.5–5.2) years in the mediastinitis and 2.8 (range 0.7–4.6) years in the control group (*p* = 0.87) (Figure 1). Evaluation of the endpoints was performed *via* coronary CT angiography. The evaluation of the images was performed by two independent radiologists blinded to the status of the patients and exposition to mediastinitis. Surgical technique for the CABG operation, surgical revision of mediastinitis and anesthesia were performed as previous described (1, 6). All cases of cardiac surgery were performed with cardiopulmonary bypass machine and no Off-Pump method was used.

### NT-proBNP, TnT, and CRP analyses

The levels of TnT were determined by a third-generation high-sensitivity assay on an Elecsys (Roche Diagnostic, Basel Switzerland), with a level of detection of 0.01 μg/L. CRP concentrations were measured with a chemiluminescent enzyme-labeled immunometric high-sensitivity assay (Immulite CRP; Diagnostic Products Corp), with a limit of detection of 0.1 mg/L. Serum NT-proBNP was determined with a sandwich immunoassay on an Elecsys 2010 (Roche Diagnostics).

### Statistical methods

The IBM SPSS 20.0 software (IBM Corporation, Armonk, New York) was used for data management and analysis. Data are presented as mean ± SD for continuous variables and as percentages for categorical variables. NT-proBNP, CRP, and TnT were not normally distributed and were log-transformed. Differences in the level of NT-proBNP, CRP, and TnT between mediastinitis patients and controls were assessed using a non-parametric statistic (Mann Whitney U test) as the distribution of those markers were not Gaussian and very skewed (18). Association between the presence of mediastinits and the three markers was done *via* the multivariate linear regression models to control for the variables with confounding effect in the relationship between presence of mediastinitis and outcome. All markers were logged when considered as outcomes in the multivariate model (19). *P* < 0.05 (two-sided) were considered statistically significant.

## Results

This was a cohort study of exposed (mediastinitis) and non-exposed (random controls without mediastinitis) patients chosen from the same source population. Baseline characteristics are presented in Table 1. There was no difference in age, gender, New York Heart Association (NYHA) functional class, and left ventricular ejection fraction (EF) between the groups, but body mass index (BMI) was significantly higher in patients with mediastinitis. Patients with mediastinitis had also higher burden of previous MI and diabetes (Table 1). The need for blood transfusion in relation to the CABG procedure was also significantly higher in patients with mediastinitis (*p* = 0.03). In contrast to these differences, there was no difference in number of stenotic coronary arteries with 2.7 (±0.5) in the mediastinitis group and 2.8 (±0.4) in the control group (mean ± SD, *p* = 0.71).

**Table 1:** Clinical profile of patients with mediastinitis and random controls without mediastinitis.

	**Mediastinitis** **(*n* = 41)**	**Controls** **(*n* = 41)**	***p*-value**
**Preoperative**			
Age (years)	67.0 (±9.9)	64.0 (±9.9)	0.30
Male gender (%)	87.8	78.0	0.13
Body mass index (kg/m^2^)	30.1 (±4.0)	27.0 (±3.9)	< 0.01
Hypertension (%)	64	61	0.60
NYHA (III–IV) (%)	68.2	49.1	0.09
Ejection fraction (%)	60 (±14.5)	66.2 (±9.9)	0.29
Left main stenosis (%)	31.7	26.8	0.28
Emergency surgery (%)	39.0	24.4	0.12
Unstable angina (%)	43.9	24.4	0.05
Previous myocardial infarction (%)	65.9	34.1	< 0.01
Preoperative atrial fibrillation (%)	17.1	9.8	0.52
Chronic obstructive pulmonary disease (%)	22.0	14.6	0.28
Diabetes (%)	41.5	7.3	< 0.01
**Operative**			
Aortic cross-clamp time (min)	36.1 (±15.6)	32.5 (±13.9)	0.32
Cardio-pulmonary bypass time (min)	60.8 (±28.5)	50.6 (±18.5)	0.12
**Postoperative**			
Mediastinal drainage (ml)	740 (±422)	695 (±278)	0.77
Blood transfusion (units)	1.2 (±2.6)	0.2 (±0.8)	0.03

### Differences in NT-proBNP, TnT, and CRP level between the study groups

**Figure 2:**
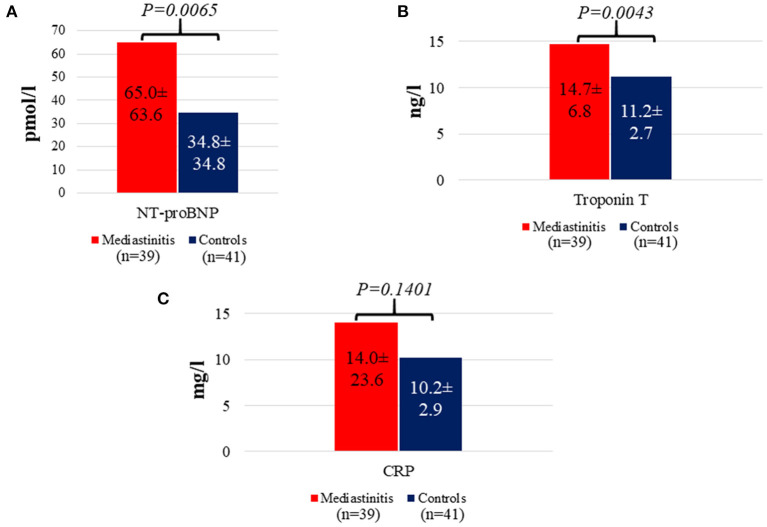
Distribution of NT-proBNP **(A)**, Troponin T **(B)**, and CRP concentrations **(C)** in patients with mediastinitis versus controls. Date are presented here as mean ± SD.

Patients with mediastinitis following CABG had significantly higher levels of NT-proBNP as compared with those without this complication 65.0 (±63.6) pmol/l vs. 34.8 (±34.8) pmol/l (mean ± SD, *p* = 0.007) (Figure 2). A similar pattern was seen for TnT with the highest levels in those with mediastinitis 14.7 (±6.8) ng/l vs. 11.2 (±2.7) ng/l (mean ± SD, *p* = 0.004), but not for CRP (*p* = 0.14) (Figure 2).

### Multivariate analysis controlling for confounders

Table 2 summarizes the results of the univariate and multivariate linear regression models. In separate models after controlling for the potential confounders (i.e., previous MI, age, and BMI), the presence of mediastinitis was associated with higher log NT-proBNP (*p* = 0.02) and log TnT (*p* = 0.04).

**Table 2:** Univariate and multivariable-adjusted effect of mediastinits on log NT-proBNP and log troponin-T.

	**Coefficient**	**SE (coefficient)**	**Statistics-Z**	***p*-value**
**Endpoint log NT-proBNP**				
**Univariate**				
Mediastinitis (yes/no)	0.6845	0.2251	3.04	0.030
**Multivariable**				
Mediastinitis (yes/no)	0.5200	0.2180	2.39	0.020
Previous MI	0.4916	0.2028	2.42	0.018
Age	0.0465	0.0099	4.71	< 0.001
BMI	−0.0403	0.0251	−1.61	0.090
**Endpoint log troponin-T**				
**Univariate**				
Mediastinitis (yes/no)	0.1962	0.6715	2.92	0.005
**Multivariable**				
Mediastinitis	0.1483	0.0710	2.09	0.040
Previous MI	0.1470	0.0661	2.22	0.029
Age	0.0090	0.0032	2.80	0.007
BMI	−0.0058	0.0816	−0.71	0.010

BMI, body mass index; MI, myocardial infarction.

## Discussion

In the present study we showed that patients with VAC-treated mediastinitis after CABG, had significantly higher serum levels of NT-proBNP and TnT, but not of CRP, compared with patients without mediastinitis even 2.7 years (median) following CABG. Cardiac biomarkers like NT-proBNP and TnT are useful for prediction of cardiac events and mortality (7–14), and raised levels in the mediastinitis group may at least partly explain the observed decreased long-term survival in this group of patients.

Mediastinitis is a severe complication after CABG, and is associated with increased risk of early and late cardiovascular death (1). In a prospective study of 5,185 patients undergoing cardiac surgery, 41 patients had mediastinal infection. Among these, 16 patients had isolated CABG and 8 combined with valve intervention (20). Mediastinal infection after cardiac operation was associated with longer hospital stay, readmissions, and death. However, the frequency of graft obstruction and its association with prognosis was not studied. Based on our previous observations in this cohort (6), we hypothesized that inflammation pathway may be a major contributor to the pathogenesis of atherosclerotic obstruction of the IMA graft, and this might explain why postoperative mediastinitis is a major contributor of reduced long-term survival after CABG.

Levels of NT-proBNP rise with increasing degree of cardiac wall stress secondary to ventricular enlargement and maladaptive cardiac remodeling seen in patients with HF and after MI (21). Importantly, however, NT-proBNP is secreted in response to raised intra-cardiac pressure and wall stress irrespective of the underlying cause (7–10). TnT is a sensitive marker of cardiomyocyte damage following necrosis or apoptosis, and raised levels have been shown to provide important prognostic information in various cardiovascular disorders including MI, stable CAD and HF (11, 12). In the present study, we show that patients with mediastinitis following CABG have elevated levels of NT-proBNP and TnT more than 2 years following CABG. These findings suggest that patients with mediastinitis may have persistent myocardial damage and impaired cardiac function, potentially contributing to the poorer long-term prognosis in these patients that also include increased occurrence of cardiac death (1).

Although the exact reasons for the increased levels of NT-proBNP and TnT in patients with mediastinitis remain unknown, a possible explanation may be the significantly higher prevalence of cardiometabolic risk factors in the mediastinitis group. It is well-established that metabolic syndrome leads to structural and functional left ventricular remodeling, preclinical or clinical systolic and/or diastolic dysfunction and fibrosis (22). However, elevated levels of TnT and NT-proBNP were also found after adjusting for BMI, suggesting that our findings may not merely reflect an increased prevalence of cardiometabolic risk in patients with mediastinitis. It is also important to note that NT-proBNP has been seen to be affected by other factors such as anemia (23) and renal failure (24) that was not adjusted for in the present study. Furthermore, the prevalence of prior MI was as twice as common in patients with mediastinitis compared with controls. Hence, silent/residual coronary ischemia, particularly due to microvascular disease, in patients with mediastinitis may also be a possible reason for increased TnT. However, all patients were clinically stable and asymptomatic, and underlying silent coronary ischemia was not assessed by stress echocardiography or other imaging modalities. Nonetheless, this highlights the importance of optimal cardioprotective medications including statin and anti-angina and antihypertensive medications following CABG surgery.

Persistent mediastinitis *per se* as an inflammatory condition could potentially be a contributing factor, but the lack of difference in CRP levels between the two groups, makes this possibility unlikely. However, low-grade myocardial inflammation that is not reflected by systemic CRP levels cannot be excluded. Moreover, mediastinitis may lead to an accelerated coronary ischemia and artery spasm, potentially contributing to ischemic cardiomyopathy. Also, the presence of mediastinitis in the early period following CABG, increases the risk of IMA-graft obstruction, but not saphenous vein grafts (SVG) (6). The mechanism of mediastinitis-induced IMA failure, which in turn could induce myocardial dysfunction, may be mechanical damage due to repetitive sternal revision or inflammatory reaction to the IMA with subsequent occlusion. Whatever the mechanisms, our findings suggest that mediastinitis following CABG may induce myocardial dysfunction that could be evident even more than 2 years following CABG surgery.

The present study has some limitations. First, our study groups are relatively small with some additional methodological limitations such as the retrospective design and lack of long-term outcome data; however we believe our results are of topical interest, hypothesis generating and should trigger lager prospective studies with longer follow-up to investigate the association of post-CABG mediastinitis with cardiac biomarkers as well as with serum biomarkers of inflammation. Second, serum biomarkers were retrospectively collected and the study lacks some relevant data. Therefore, our conclusions should be interpreted cautiously. Third, the lack of data on mortality and morbidity, including cardiac related hospitalizations and the incidence of recurrent angina. Fourth, only baseline left ventricular EF by echocardiography was included. Data on global longitudinal strain derived by speckle-tracking echocardiography or diastolic dysfunction were not available. Similarly, echocardiographic data during follow-up was not collected. Fifth, cardiac magnetic resonance (CMR) imaging is a gold standard for assessment of persistent myocardial inflammation, subendocardial injury and scar and fibrosis. Our findings suggest that mediastinitis following CABG surgery could induce a degree of myocardial dysfunction potentially contributing to the increase in short- and long-term mortality in these patients. However, CMR was not included in the present study. Larger prospective studies in the future that also include echocardiography and CMR should examine whether elevated TnT and NT-proBNP concentration could be used as prognostic markers for long term survival after CABG.

## Data availability statement

The original contributions presented in the study are included in the article/supplementary material, further inquiries can be directed to the corresponding author.

## Ethics statement

The study was approved by the Regional Medical Ethics Committee, and represents a multi-center collaboration between Oslo University Hospital, Rikshospitalet, Feiring Heart Clinic, Oslo Heart Center and Akershus University Hospital. The institutions maintain the same diagnostic criteria and treatment with active and prospective epidemiologic surveillance of hospital infections. Written informed consent was obtained from all participants.

## Author contributions

IR: data collection, project administration, and original draft. IR, PA, and SS: conceptualization, formal analysis, methodology, supervision, validation, visualization, and writing. IR and SS: literature search. RL, OR, TU, and SR: review and editing. PA and SS: critically revised the manuscript. All authors read and approved the final version before submission.
